# Community of inquiry presences and undergraduates’ online learning outcomes: self-regulated learning as a pathway and knowledge-seeking intention as a boundary condition

**DOI:** 10.3389/fpsyg.2026.1895876

**Published:** 2026-08-19

**Authors:** Junfei Li, Jinyan Huang, Jai Kumar

**Affiliations:** 1 Shanghai Dianji University, Shanghai, China; 2 Jiangsu University, Zhenjiang, China; 3 Xiangnan University, Chenzhou, China

**Keywords:** community of inquiry, knowledge-seeking intention, moderated mediation, online learning satisfaction, self-regulated learning

## Abstract

This cross-sectional study examined how teaching presence (TP), social presence (SP), and cognitive presence (CP) were associated with undergraduates’ perceived task performance (PTP) and online learning satisfaction (OLS), with self-regulated learning (SRL) as a mediator and knowledge-seeking intention (KSI) as a first-stage moderator. Survey responses from 726 students enrolled at multiple Chinese universities were analyzed using partial least squares structural equation modeling and conditional process analysis. SRL was positively associated with both PTP and OLS. TP and CP were positively associated with SRL, whereas SP showed a significant negative partial coefficient after the three presences were modeled simultaneously. The indirect associations through SRL were positive for TP and CP but negative for SP. KSI significantly conditioned the TP- and SP-based indirect associations but not the CP-based indirect associations. The negative SP coefficient should not be interpreted as evidence that social presence is harmful; high inter-construct correlations, a marginal Fornell–Larcker violation, and elevated full-collinearity variance inflation factors indicate possible construct overlap, suppression, and common method variance. The findings therefore support a cautious interpretation of SRL as a potential pathway and KSI as a boundary condition, while highlighting the need for longitudinal, multi-source, and measurement-refined replication.

## Introduction

Online and blended learning are now routine components of higher education. Their effectiveness depends not only on access to digital platforms but also on whether students experience coherent instructional guidance, meaningful interaction, sustained inquiry, and sufficient autonomy to manage their own learning (Getenet et al., 2024; Shea et al., 2022). Identifying the processes through which online learning environments are associated with students’ performance and satisfaction is therefore important for both theory and course design (Ros, 2024; Zalazar-Jaime et al., 2023).

The Community of Inquiry (CoI) framework conceptualizes a productive educational experience as the intersection of teaching presence, social presence, and cognitive presence (Garrison et al., 1999, 2001). Teaching presence concerns instructional design, facilitation, and direction; social presence concerns the capacity to participate as a socially and affectively recognizable member of a learning community; and cognitive presence concerns constructing and confirming meaning through reflection and discourse (Garrison et al., 1999; Shea et al., 2022). Although the three presences are analytically distinguishable, they are theoretically interdependent and often highly correlated in survey research (Garrison and Arbaugh, 2007; Kozan and Caskurlu, 2018; Nasir and Ngah, 2022).

Prior research generally links stronger CoI presences with perceived learning, engagement, persistence, and course satisfaction, but the size and consistency of these associations vary across samples and course formats (Arbaugh et al., 2008; Akyol and Garrison, 2008; Joo et al., 2011; Richardson et al., 2017). One reason may be that the presences are environmental affordances rather than learning outcomes in themselves. Students must translate structure, interaction, and inquiry opportunities into goals, strategies, monitoring, help seeking, and persistence. Self-regulated learning (SRL) is therefore a plausible pathway between the CoI presences and learning outcomes (Huang et al., 2019; Lam, 2015; Barnard et al., 2008; Cho et al., 2017; Shea and Bidjerano, 2012).

However, students differ in whether they actively seek knowledge from readings, writing, discussion, instructors, and peers. Knowledge-seeking intention (KSI) captures this action-oriented motivational orientation (Khuram et al., 2021). In contrast to broad motivation, KSI is proximal to the use of the very resources represented by teaching and social presence. It may therefore change the strength with which a learning environment is translated into SRL. A student with strong KSI may independently search for explanations, ask questions, and use feedback, whereas a student with weak KSI may rely more heavily on externally provided structure. This study consequently treats KSI as a boundary condition rather than as another component of SRL.

The study addresses three questions: (1) How are TP, SP, and CP associated with SRL, PTP, and OLS? (2) Does SRL statistically mediate the associations between the three presences and the two outcomes? (3) Do the indirect associations vary as a function of KSI? By examining the three presences in one model, the study also provides an opportunity to distinguish their unique partial associations from their substantial shared variance. Because the design is cross-sectional and all variables are self-reported, the analysis concerns statistical associations and conditional indirect effects, not demonstrated causal mechanisms.

## Literature review and hypotheses

### The community of inquiry presences

Teaching presence provides the organizational and pedagogical conditions for productive online learning. Clear objectives, aligned activities, facilitation, and timely feedback can reduce ambiguity and help students allocate effort (Arbaugh et al., 2008; Zhang et al., 2016). Teaching presence is therefore expected to support SRL by making goals and performance standards visible. At the same time, very strong instructor control may reduce the need for independent regulation, suggesting that the association can depend on learner motivation and course design (Akyol and Garrison, 2008).

Social presence supports belonging, open communication, and the interpersonal trust needed for collaborative inquiry(Garrison, 2009). Meta-analytic evidence indicates positive associations with satisfaction and perceived learning (Richardson et al., 2017). Social presence can support SRL through peer modeling, accountability, help seeking, and co-regulation (Shea et al., 2013; Zheng et al., 2023). Yet social interaction can also be superficial, distracting, or demanding, and its unique coefficient may change when teaching and cognitive presences are entered simultaneously. Accordingly, both substantive and statistical overlap among the presences must be considered when interpreting partial coefficients.

Cognitive presence reflects the progression from triggering problems to exploration, integration, and application. Reflection and sustained discourse are closely related to metacognitive monitoring and strategic regulation, making CP a theoretically strong antecedent of SRL (Garrison et al., 2001; Al Mamun and Lawrie, 2024). Empirical studies commonly link CP to perceived learning and satisfaction, although its discriminant separation from SRL can be challenging when both are measured with self-report items concerning reflection and meaning making (Hu et al., 2023).

Taken together, the three presences are expected to support SRL through complementary mechanisms. TP reduces uncertainty by communicating goals, sequencing activities, and providing feedback, thereby supplying external standards that students can internalize in planning and monitoring. SP creates a relational climate in which learners can ask for help, observe peers’ strategies, and participate in co-regulation. CP requires learners to identify problems, integrate evidence, evaluate explanations, and apply knowledge, processes that directly engage metacognitive monitoring and strategic control. Empirical CoI research has likewise positioned teaching and social presence as conditions that help sustain cognitive engagement, and learning-presence research has argued that self-regulatory processes explain how learners actively enact these environmental conditions (Garrison et al., 2010; Garrison and Cleveland-Innes, 2005; Shea and Bidjerano, 2010). Accordingly, TP, SP, and CP are each hypothesized to be positively associated with SRL, while their unique coefficients are interpreted in light of their substantial interdependence.

### Self-regulated learning as a pathway

SRL refers to learners’ active management of goals, time, strategies, monitoring, help seeking, and self-evaluation (Pintrich, 2004; Zimmerman, 2008). Online learning increases the importance of these processes because students often have greater control over when, where, and how they engage. SRL has been associated with achievement, perceived learning, persistence, and satisfaction in online settings (Barnard et al., 2008; Li, 2019).

The CoI presences can be understood as resources that support regulation. Teaching presence supplies goals, sequencing, standards, and feedback; social presence supplies relational support and opportunities for help seeking and co-regulation; cognitive presence supplies inquiry structures that prompt monitoring, integration, and application. SRL, in turn, may connect these resources with perceived task accomplishment and satisfaction. This pathway interpretation is consistent with prior work positioning learning presence or self-regulation as an extension of the CoI model (Shea and Bidjerano, 2012; Hu et al., 2023).

The proposed pathway also specifies why SRL should be associated with the two outcomes. Students who set goals, manage time, select strategies, monitor understanding, and seek assistance are better positioned to complete tasks efficiently and to experience progress and control in online courses. Systematic and empirical evidence links online SRL with academic achievement and course satisfaction, while studies of e-learning success identify learner self-management and interaction as important predictors of perceived learning and satisfaction (Broadbent and Poon, 2015; Eom et al., 2006; Kuo et al., 2014). Thus, the presences are not assumed to produce outcomes automatically; rather, they provide instructional, interpersonal, and inquiry resources that students must convert into regulated action. This resource-to-regulation-to-outcome sequence provides the rationale for testing SRL as a mediator.

### Knowledge-seeking intention modeled as a boundary condition

KSI is the intention to acquire knowledge through courses, reading, writing, discussion, and interaction (Lai et al., 2014; Tsai and Kang, 2019). It is related to motivation but is narrower and more behaviorally proximal. SRL describes how students manage learning; KSI describes whether they are inclined to pursue additional understanding and information. The constructs should therefore be related but not interchangeable.

KSI is singled out as a moderator because the usefulness of CoI resources depends on students’ willingness to use them. Students with stronger KSI may transform instructor feedback and peer interaction into strategic action more readily (Kankanhalli, 2002; Tsai and Kang, 2019). Alternatively, they may require less external structure because they already search independently, which could attenuate the association between teaching presence and SRL (Khuram et al., 2021). KSI may also buffer unproductive social experiences by directing interaction toward academic purposes. These competing possibilities make KSI theoretically appropriate as a boundary condition and justify testing moderation rather than assuming uniformly stronger effects.

KSI is not proposed as the only possible moderator. Prior knowledge, self-efficacy, digital literacy, epistemic beliefs, and cultural expectations may also condition CoI relationships (Huang et al., 2019). The present model isolates KSI to test one theoretically proximal motivational mechanism while maintaining a manageable model.

Modeling KSI on the first-stage presence-to-SRL paths follows this logic. KSI may alter how strongly students convert available CoI resources into regulatory behavior: high-KSI learners may use feedback, discussion, and information sources more deliberately, but they may also substitute self-initiated searching for instructor-provided structure. The same motivational orientation could channel social interaction toward academic help seeking and reduce the regulatory costs of unfocused communication. Because CP already contains exploration, integration, and application, its association with SRL may be less contingent on KSI than the more externally supplied TP and SP resources. The moderation hypotheses are therefore nondirectional with respect to the magnitude of the conditional indirect effects, but they test whether the indirect associations vary significantly across levels of KSI.

### Learning outcomes

Perceived task performance (PTP) refers to students’ self-evaluations of planning, prioritizing, maintaining outcome focus, distinguishing central from peripheral issues, and completing learning tasks efficiently. It is not an objective grade or externally scored performance measure. Online learning satisfaction (OLS) refers to satisfaction with peer interaction, the learning environment, discussion, collaboration, and course delivery (Kuo et al., 2013; Rockinson-Szapkiw et al., 2016). Both outcomes are relevant to online course functioning but should be interpreted as perceptions.

PTP and OLS capture related but distinct dimensions of online-learning success. PTP is a cognitive-behavioral self-appraisal of whether learners organize and complete required work effectively, whereas OLS is an affective evaluation of the quality and acceptability of the learning experience. Prior online-learning models treat perceived learning or performance and satisfaction as complementary outcomes because students can judge themselves as productive without being satisfied, or be satisfied without demonstrating efficient task accomplishment (Eom and Ashill, 2016; Eom et al., 2006; Rockinson-Szapkiw et al., 2016). Examining both outcomes therefore provides a broader test of whether SRL is associated with what students believe they accomplish and how positively they evaluate the course.

SRL is expected to relate positively to PTP because planning, prioritization, time management, monitoring, and adaptive help seeking should improve task focus and completion efficiency. It is expected to relate positively to OLS because effective regulation reduces uncertainty, supports a sense of control, and increases the likelihood that learners experience successful progress. These expectations are consistent with evidence that self-regulatory strategies predict achievement, perceived learning, and satisfaction in online higher education (Broadbent and Poon, 2015; Kuo et al., 2014; Li, 2019).

Combining these relationships yields the mediation hypotheses. TP should contribute to PTP and OLS through SRL by clarifying goals and standards; SP should contribute through help seeking, peer modeling, and co-regulation; and CP should contribute through reflection, integration, and application. Prior CoI studies and meta-analyses report positive associations of teaching and social presence with perceived learning and satisfaction, while cognitive-presence research links sustained inquiry with deeper learning outcomes (Akyol and Garrison, 2011; Caskurlu et al., 2020; Richardson et al., 2017). The present model therefore tests whether SRL statistically carries these presence-outcome associations and whether KSI conditions the first stage of each indirect association.

**Figure 1:**
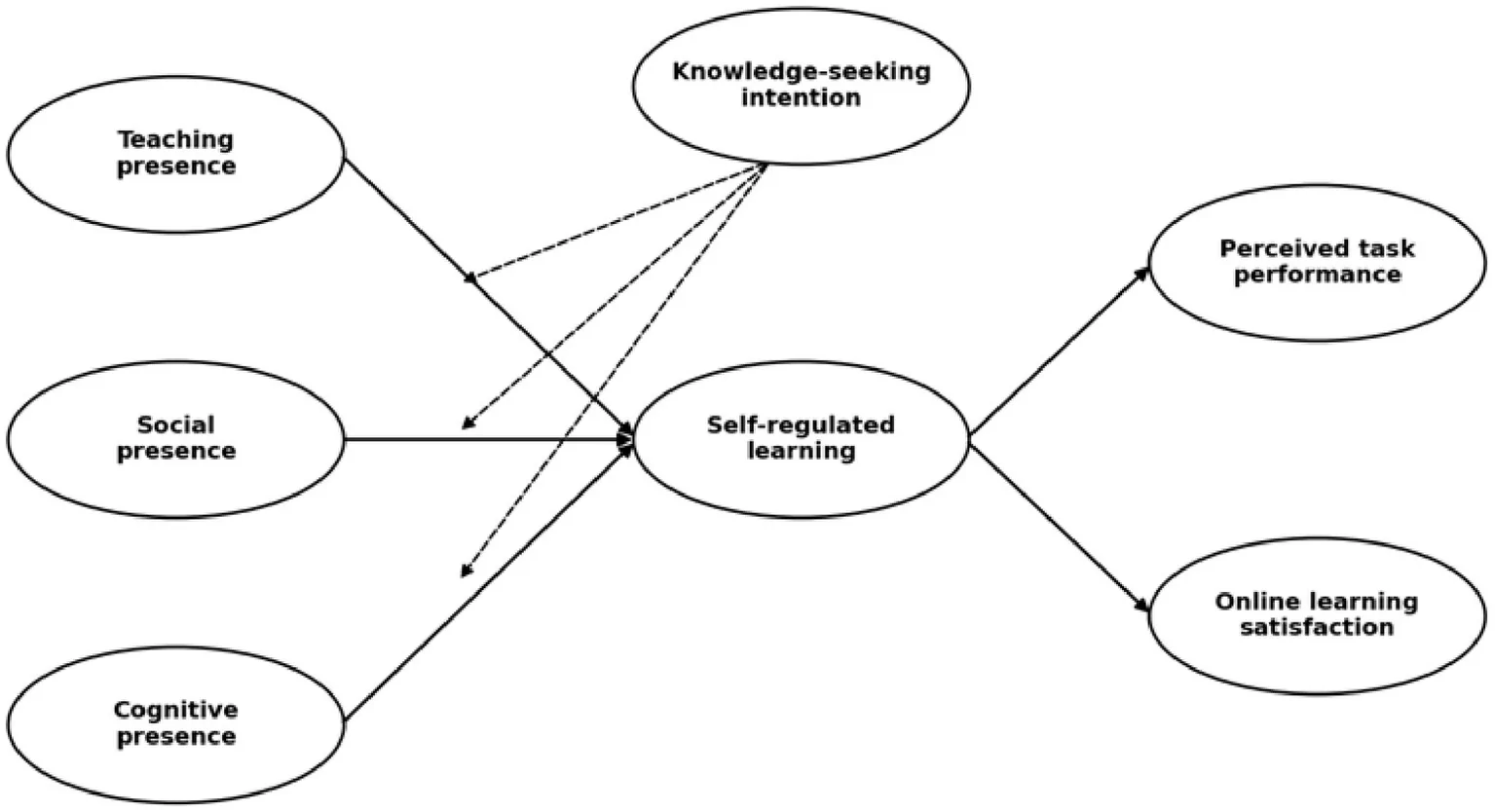
Hypothesized moderated-mediation model. Dashed arrows indicate moderation of the presence → SRL paths by KSI.

Based on the preceding reasoning, the study proposes direct, mediated, and moderated-mediation hypotheses (see Figure 1). Directional hypotheses are evaluated according to both statistical significance and the sign of the coefficient; a significant result in the opposite direction is treated as not supporting the hypothesis.

*H1:* SRL is positively associated with PTP.

*H2:* SRL is positively associated with OLS.

*H3:* TP is positively associated with SRL.

*H4:* SP is positively associated with SRL.

*H5:* CP is positively associated with SRL.

*H6:* SRL carries a positive indirect association between TP and PTP.

*H7:* SRL carries a positive indirect association between TP and OLS.

*H8:* SRL carries a positive indirect association between SP and PTP.

*H9:* SRL carries a positive indirect association between SP and OLS.

*H10:* SRL carries a positive indirect association between CP and PTP.

*H11:* SRL carries a positive indirect association between CP and OLS.

*H12:* KSI moderates the indirect association between TP and PTP through SRL.

*H13:* KSI moderates the indirect association between TP and OLS through SRL.

*H14:* KSI moderates the indirect association between SP and PTP through SRL.

*H15:* KSI moderates the indirect association between SP and OLS through SRL.

*H16:* KSI moderates the indirect association between CP and PTP through SRL.

*H17:* KSI moderates the indirect association between CP and OLS through SRL.

## Methodology

### Research design

The study used a quantitative, cross-sectional, multi-institutional survey design. The design permits estimation of associations among the constructs at one point in time but cannot establish temporal order or causality.

### Participants, recruitment, and data collection

The survey was administered between March and April 2024 to undergraduate students enrolled in online or blended courses at multiple universities in China. The sample size was determined based on Nunnally’s (1978) criterion, which suggests that a sufficiently large sample size is required to achieve reliable and valid results. After data screening, 726 responses were retained for analysis.

Participants completed the questionnaire electronically. The survey began with an information and consent page, followed by demographic questions and construct-specific item blocks. The administered questionnaire contained 47 substantive items. Forty-two items were used in the seven-construct model reported in this article: TP (7), SP (6), CP (6), SRL (7), KSI (5), PTP (5), and OLS (6). Five additional positive-affect items were administered as a supplementary block but were not assigned to PTP or OLS and were excluded from every analysis reported here. All items used a five-point agreement scale from 1 (strongly disagree) to 5 (strongly agree). The original administered item numbers and their analytic assignments are documented in Table A1 and Appendix A.

### Instrument adaptation and content validation

The English-language source items were adapted for the Chinese higher-education context. A careful translation and cultural validation process was undertaken to ensure their suitability for the Chinese context. Initially, the instruments were translated from English to Chinese by two bilingual experts to ensure conceptual equivalence. To further confirm the cultural relevance, the translated scales were reviewed by a panel of four experts in education and online learning within the Chinese context. This process ensured that the items were culturally appropriate and meaningful to the participants of this study.

### Instrument administration and analytic coding

The original questionnaire, item-coding key, and analysis specification were checked together. The five planning and performance items originally numbered 37–41 were coded as PTP, and the six satisfaction items originally numbered 42–47 were coded as OLS. The five positive-affect items originally numbered 32–36 were collected as supplementary items and were not included in the focal model. Table A1 provides the complete item-to-construct coding map.

### Measures

Teaching presence (TP) was measured with seven items concerning objectives, schedules, explanation, exploration, participation, feedback, and task completion, adapted from CoI measures (Arbaugh et al., 2008; Hu et al., 2023; Shea and Bidjerano, 2010; Swan et al., 2008).

Social presence (SP) was measured with six items concerning instructor immediacy, belonging, online social interaction, free communication, conversational comfort, and forming impressions of course participants (Arbaugh et al., 2008; Hu et al., 2023; Shea and Bidjerano, 2010; Swan et al., 2008).

Cognitive presence (CP) was measured with six items concerning integrating information, reflection, application, exploration, use of information sources, and appreciation of multiple perspectives (Akyol and Garrison, 2008; Garrison et al., 2001).

Self-regulated learning (SRL) was measured with seven items concerning standards, goals, schedules, task completion, peer help seeking, instructor help seeking, and self-evaluation (Barnard et al., 2008; Zimmerman, 2008).

Knowledge-seeking intention (KSI) was measured with five items concerning intentions to acquire knowledge through the degree program, courses, academic reading, academic writing, and academic discussion (Khuram et al., 2021; Lai et al., 2014; Tsai and Kang, 2019).

Perceived task performance (PTP) was measured with five items concerning planning tasks, setting priorities, focusing on required outcomes, distinguishing main from peripheral issues, and completing tasks efficiently. The construct is labeled perceived task performance because it is self-reported and does not represent objective grades or externally observed task performance (Li et al., 2023; Widyastuti and Hidayat, 2018).

Online learning satisfaction (OLS) was measured with six items concerning satisfaction with peer interaction, the learning environment, discussion, peer relationships, collaboration, and overall course delivery (Kuo et al., 2013; Rockinson-Szapkiw et al., 2016).

### Ethical considerations

This study involving human participants was reviewed and approved by the Research Ethical Review Board of the corresponding author’s research organization on January 12, 2024 (approval number ERCEA2401). Participation was described as voluntary. Students were informed of the study purpose, confidentiality protections, the right to decline or withdraw, and the intended use of aggregated data. No directly identifying information was reported in the analytical dataset.

### Data analysis

Descriptive statistics and internal-consistency estimates were produced in SPSS 24. The reflective measurement model and structural model were evaluated in SmartPLS 4. Reliability was examined using Cronbach’s alpha and composite reliability (CR), and convergent validity was examined using indicator loadings and average variance extracted (AVE). Because alpha values above approximately 0.95 can indicate redundancy as well as consistency, the unusually high values were interpreted cautiously.

Discriminant validity was examined with the Fornell and Larcker (1981) criterion. The structural paths were evaluated with 5,000 bootstrap resamples. Exact *p* values are reported, and *p* = 0.000 is presented as *p* < 0.001.

For the conditional indirect effects, latent-variable scores were exported for regression-based conditional process analysis. The hypothesized first-stage moderation is equivalent to PROCESS Model 7: each CoI presence served as X, SRL as M, KSI as W, and either PTP or OLS as Y. Six models were therefore estimated, with 5,000 bootstrap samples for the indices of moderated mediation (Hayes, 2012, 2013). Conditional indirect effects with bootstrap confidence intervals were reported.

Common method and collinearity risk were evaluated using full-collinearity VIFs reconstructed from the reported latent-construct correlation matrix (Kock, 2015). Because the values are high, this diagnostic is treated as a warning rather than as proof of a specific bias source. Procedural and statistical remedies are discussed in light of Podsakoff et al. (2003).

The measurement model, structural model, indirect-effect analyses, and moderated-mediation analyses were run using the 42-item analytic coding shown in Table A1. The screened sample of 726 cases and the estimation settings described above were used. The reliability and validity estimates, structural coefficients, indirect effects, moderated-mediation indices, and hypothesis decisions to the precision displayed in Tables 1–6.

**Table 1:** Constructs reliability and convergent validity.

Construct	Cronbach’s α	CR	AVE
Teaching presence (TP)	0.957	0.965	0.797
Social presence (SP)	0.948	0.958	0.793
Cognitive presence (CP)	0.960	0.968	0.834
Knowledge seeking intention (KSI)	0.952	0.963	0.840
Self-regulated learning (SRL)	0.950	0.959	0.771
Perceived task performance (PTP)	0.952	0.963	0.839
Online learning satisfaction (OLS)	0.963	0.970	0.843

CR, composite reliability; AVE, average variance extracted.

**Table 2:** Fornell–Larcker discriminant-validity matrix.

Construct	CP	KSI	OLS	SP	SRL	PTP	TP
CP	0.**913**						
KSI	0.828	0.**917**					
OLS	0.798	0.807	0.**918**				
SP	0.862	0.777	0.819	0.**891**			
SRL	0.880	0.870	0.822	0.851	0.**878**		
PTP	0.819	0.838	0.840	0.797	0.855	0.**916**	
TP	0.797	0.773	0.738	0.771	0.760	0.767	0.893

Bold diagonal values are square roots of AVE; off-diagonal values are inter-construct correlations. The SRL–CP pair violates the criterion because 0.880 exceeds the SRL diagonal value of 0.878.

**Table 3:** Full-collinearity variance inflation factors.

Construct	VIF
CP	6.30
KSI	5.31
OLS	4.57
SP	5.28
SRL	7.44
PTP	5.29
TP	3.35

Values above 3.3 are a conservative warning for common method bias; values above 5 also indicate substantial collinearity.

**Table 4:** Structural relationships and hypothesis testing.

Hypothesis	Path	B	SE	*t*	*p*	Decision
H1	SRL → PTP	0.843	0.032	26.458	< 0.001	Supported
H2	SRL → OLS	0.844	0.026	32.430	< 0.001	Supported
H3	TP → SRL	0.447	0.180	2.486	0.013	Supported
H4	SP → SRL	−0.555	0.189	2.938	0.003	Not supported: opposite direction
H5	CP → SRL	0.476	0.145	3.293	0.001	Supported

**Table 5:** Indirect associations through SRL.

Hypothesis	Indirect path	B	SE	*t*	*p*	Decision
H6	TP → SRL → PTP	0.377	0.154	2.449	0.014	Supported
H7	TP → SRL → OLS	0.378	0.155	2.443	0.015	Supported
H8	SP → SRL → PTP	−0.468	0.161	2.903	0.004	Not supported: opposite direction
H9	SP → SRL → OLS	−0.469	0.161	2.917	0.004	Not supported: opposite direction
H10	CP → SRL → PTP	0.402	0.123	3.260	0.001	Supported
H11	CP → SRL → OLS	0.402	0.080	3.505	< 0.001	Supported

A significant effect in the opposite direction does not support a directional hypothesis.

**Table 6:** Indices of moderated mediation.

Hypothesis	Conditional indirect path	Index	SE	*t*	*p*	Decision
H12	TP → SRL → PTP	−0.101	0.039	2.563	0.010	Supported
H13	TP → SRL → OLS	−0.101	0.040	2.562	0.010	Supported
H14	SP → SRL → PTP	0.174	0.041	4.220	<0.001	Supported
H15	SP → SRL → OLS	0.174	0.041	4.238	<0.001	Supported
H16	CP → SRL → PTP	−0.039	0.032	1.218	0.223	Not supported
H17	CP → SRL → OLS	−0.039	0.032	1.240	0.215	Not supported

## Results

### Verification of the analytic coding

The verification and analysis confirmed that all results reported below are based exclusively on the 42 items assigned to TP, SP, CP, SRL, KSI, PTP, and OLS in Table A1. The five supplementary positive-affect items were not used to form a construct score, latent variable, indirect effect, or interaction term in any analysis reported in this study.

### Measurement model

Table 1 presents internal consistency and convergent validity. Cronbach’s alpha ranged from 0.948 to 0.963, CR ranged from 0.958 to 0.970, and AVE ranged from 0.771 to 0.843. These values exceed conventional minimum criteria. However, the uniformly high reliability estimates may also reflect semantically similar items and block presentation, so they should not be interpreted as evidence of validity by themselves. The reported SRMR was 0.063 and NFI was 0.87; these are treated as supplementary descriptors.

Table 2 displays the Fornell–Larcker matrix. The bold diagonal entries are the square roots of AVE recalculated from Table 1. The SRL diagonal (0.878) is slightly lower than the SRL–CP correlation (0.880), so the criterion is not fully satisfied.

### Common method and collinearity diagnostic

The full-collinearity VIF values are shown in Table 3. All exceed the conservative 3.3 criterion, and five exceed 5.0. These results indicate that common method variance, construct overlap, and multicollinearity cannot be dismissed. They are especially relevant to the unexpected negative SP coefficient and the very strong SRL–outcome associations.

### Structural model

**Figure 2:**
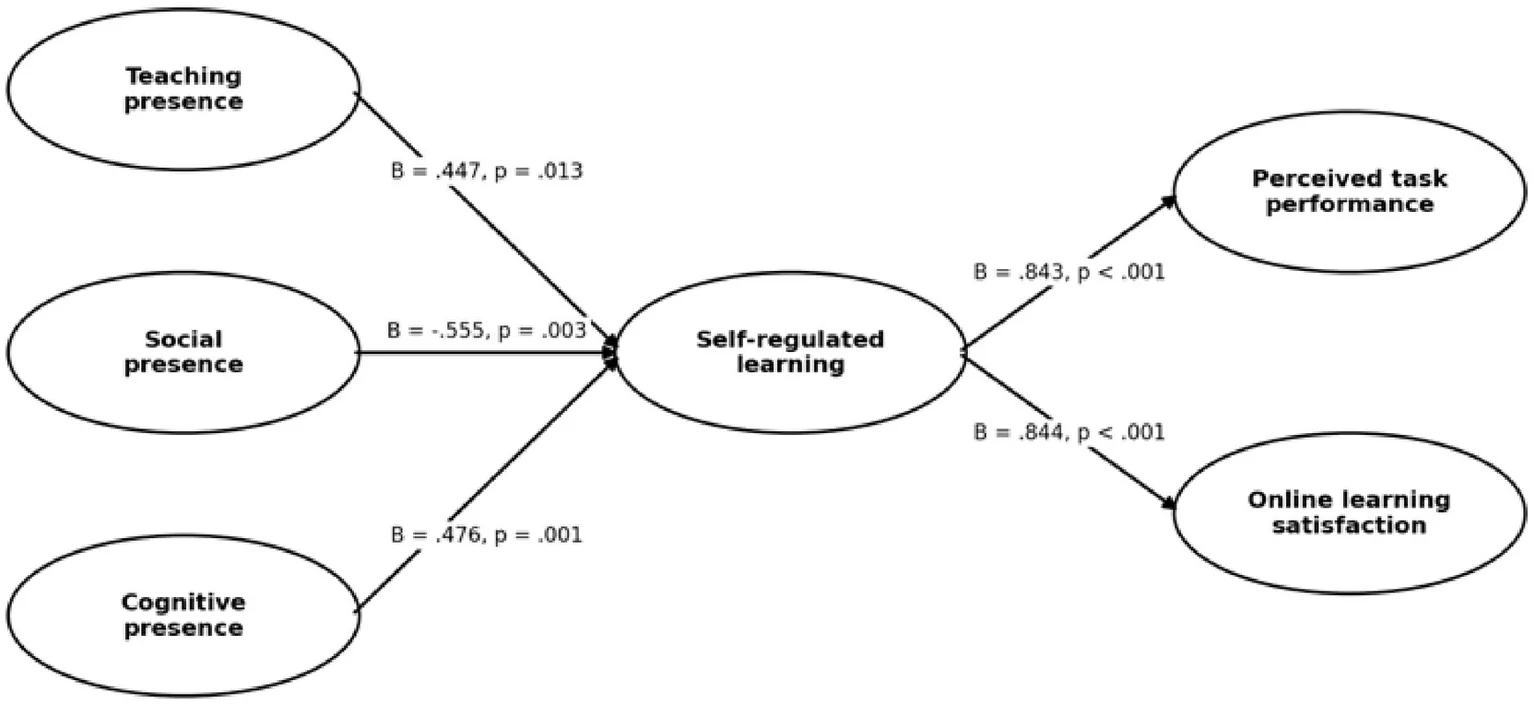
Reconciled structural coefficients.

The original hypothesis-testing tables reported R^2^ = 0.732 for PTP and R^2^ = 0.675 for OLS. Table 4 presents the internally coherent structural coefficients used in the mediation calculations. SRL was positively associated with PTP and OLS, supporting H1 and H2. TP and CP were positively associated with SRL, supporting H3 and H5. SP had a significant negative coefficient, so H4 was not supported because the result was opposite to the hypothesized positive direction (see Figure 2).

### Indirect associations through SRL

The indirect-effect estimates in Table 5 are numerically consistent with the structural coefficients in Table 4. The TP- and CP-based indirect associations were positive. The SP-based indirect associations were significant but negative; therefore, H8 and H9 were not supported as directional positive hypotheses.

### Moderated mediation

Table 6 reports the indices of moderated mediation from the original analysis. KSI significantly conditioned the TP- and SP-based indirect associations for both outcomes, supporting H12–H15. The indices involving CP were not significant, so H16 and H17 were not supported. The negative TP indices indicate attenuation of the positive TP indirect association as KSI increases. The positive SP indices indicate that stronger KSI buffers the negative SP indirect association. To probe the significant indices, conditional indirect effects were evaluated at low (−1 SD), mean (0), and high (+1 SD) levels of standardized KSI (see Table 7). The TP-based indirect associations decreased as KSI increased, whereas the SP-based indirect associations became less negative.

**Table 7:** Conditional indirect effects at low, mean, and high knowledge-seeking intention.

Conditional indirect path	KSI level	Estimate	95% Confidence intervals
TP → SRL → PTP	Low (−1 SD)	0.478	[0.167, 0.789]
	Mean	0.377	[0.075, 0.679]
	High (+1 SD)	0.276	[−0.035, 0.587]
TP → SRL → OLS	Low (−1 SD)	0.479	[0.165, 0.793]
	Mean	0.378	[0.074, 0.682]
	High (+1 SD)	0.277	[−0.037, 0.591]
SP → SRL → PTP	Low (−1 SD)	−0.642	[−0.968, −0.316]
	Mean	−0.468	[−0.784, −0.152]
	High (+1 SD)	−0.294	[−0.620, 0.032]
SP → SRL → OLS	Low (−1 SD)	−0.643	[−0.969, −0.317]
	Mean	−0.469	[−0.785, −0.153]
	High (+1 SD)	−0.295	[−0.621, 0.031]

KSI was standardized; low, mean, and high values correspond to −1 SD, the mean, and +1 SD.

As shown in Table 7, for the TP pathways, the conditional indirect association with PTP decreased from 0.478 at low KSI to 0.377 at mean KSI and 0.276 at high KSI. The corresponding association with OLS decreased from 0.479 to 0.378 and 0.277. The confidence intervals excluded zero at low and mean KSI but included zero at high KSI, suggesting that the positive TP-based indirect associations became weaker as KSI increased.

For the SP pathways, the conditional indirect association with PTP changed from −0.642 at low KSI to −0.468 at mean KSI and −0.294 at high KSI. The corresponding association with OLS changed from −0.643 to −0.469 and −0.295. The confidence intervals excluded zero at low and mean KSI but included zero at high KSI. This pattern suggests that stronger KSI buffered the negative SP-based indirect associations, which approached zero at higher levels of KSI.

## Discussion and conclusion

This study examined whether SRL statistically connected the CoI presences with two perceived online-learning outcomes and whether KSI conditioned those indirect associations. The results support several parts of the model, but the measurement and collinearity diagnostics require a cautious interpretation. In particular, the findings should not be described as causal, and the negative SP coefficient should not be treated as evidence that social presence is intrinsically detrimental.

### SRL and perceived learning outcomes

SRL showed strong positive associations with PTP and OLS. This finding confirms earlier research showing that online learners’ goal setting, time management, monitoring, help seeking, and self-evaluation are associated with achievement, perceived learning, and satisfaction (Barnard et al., 2008; Broadbent and Poon, 2015; Kuo et al., 2014; Li, 2019). It also supports Cho et al.’s (2017) evidence that self-regulation is closely connected to affective outcomes within online communities of inquiry. In substantive terms, students who perceive themselves as managing learning effectively also tend to report efficient task completion and a more satisfactory learning experience. However, the unusually large coefficients should not be read as stronger causal effects than those in prior studies, because common method variance, block presentation, and semantic proximity between SRL and the self-reported PTP items may have inflated the associations. Future studies should combine SRL surveys with grades, assignment quality, learning-management-system traces, or instructor ratings.

### Teaching and cognitive presence as antecedents of SRL

TP was positively associated with SRL. This result supports learning-presence research arguing that course design, facilitation, explicit standards, and feedback provide the external structure from which learners develop planning and monitoring processes (Shea and Bidjerano, 2010, 2012), and it is consistent with evidence that TP predicts engagement and is positively related to perceived learning and satisfaction (Caskurlu et al., 2020; Zhang et al., 2016). CP was also positively associated with SRL, confirming the theoretical expectation that problem exploration, integration, reflection, and application activate metacognitive monitoring and strategic engagement. This pattern agrees with earlier findings that cognitive presence is linked with deep approaches to learning and with SRL-mediated satisfaction (Akyol and Garrison, 2011; Garrison and Cleveland-Innes, 2005; Hu et al., 2023) and with recent work on scaffolding cognitive presence in self-regulated online environments (Al Mamun and Lawrie, 2024). Nevertheless, the CP-SRL correlation was 0.880 and marginally violated the Fornell-Larcker criterion. The positive CP path may therefore represent both a substantive relationship and measurement overlap. Replication should use more behaviorally distinct SRL indicators and external measures of cognitive engagement.

### Interpreting the negative social-presence coefficient

The negative SP coefficient is the most unexpected result and, at the level of the partial structural path, contradicts the dominant prior evidence. Richardson et al.’s (2017) meta-analysis found strong positive relationships of social presence with satisfaction and perceived learning, and Hu et al. (2023) reported a positive SRL-mediated link between social presence and learning satisfaction. The present zero-order correlations of SP with SRL (0.851), OLS (0.819), and PTP (0.797) are actually consistent with that literature. The contradiction emerged only after TP and CP were entered simultaneously, when the SP coefficient changed sign. This pattern is more plausibly interpreted as suppression or unstable estimation under multicollinearity than as evidence that social presence harms regulation. It is also compatible with findings that not every form of learner-learner interaction uniquely predicts satisfaction once other interactions and learner characteristics are considered (Kuo et al., 2014). A more defensible interpretation is that the model separated a shared “positive online learning environment” factor from a residual SP component that is difficult to interpret. The elevated VIFs strengthen this caution. Alternative models, such as a higher-order CoI factor, bifactor structure, or sequential TP → SP/CP → SRL model, should be compared in future work.

### The moderating role of knowledge-seeking intention

KSI significantly conditioned the TP- and SP-based indirect associations but not the CP-based indirect associations. Because prior KSI research has largely examined the intention to seek and exchange knowledge rather than its role in CoI pathways, these findings extend more than directly confirm or contradict earlier studies (Lai et al., 2014; Tsai and Kang, 2019). The negative TP index suggests substitution: as KSI increases, students may rely less on externally provided instructional structure because they independently search for explanations and resources. This interpretation is consistent with the learning-presence argument that learner agency can alter the extent to which environmental supports are translated into regulation (Shea and Bidjerano, 2012). The positive SP index suggests buffering: strong KSI may direct peer interaction toward academic help seeking and knowledge construction, thereby weakening the negative residual SP association in the multivariate model. The nonsignificant CP moderation is also theoretically plausible because CP already represents internally engaged exploration, integration, and application (Akyol and Garrison, 2011); there may be less additional variation for KSI to condition. These explanations remain tentative until the conditional effects are replicated with longitudinal or experimental data. The conditional estimates in Table 7 make these patterns concrete: from low to high KSI, the TP indirect association declined from 0.478 to 0.276 for PTP and from 0.479 to 0.277 for OLS. Over the same range, the SP indirect association changed from −0.642 to −0.294 for PTP and from −0.643 to −0.295 for OLS. Thus, KSI attenuated the TP pathway and buffered, but did not reverse, the negative residual SP pathway.

### Theoretical implications

The study contributes to CoI research by distinguishing an environmental pathway from a motivational boundary condition. SRL represents the learner processes through which instructional, social, and cognitive opportunities may be enacted, whereas KSI represents the intention to actively pursue knowledge from those opportunities. The results also show why the three presences should not be interpreted solely through separate path coefficients. Their substantial shared variance can produce unstable unique effects.

### Practical implications

For teaching presence, instructors can publish a weekly learning roadmap that connects objectives, activities, deadlines, and assessment criteria; provide short, scheduled feedback cycles; and explain how students should act on feedback rather than merely supplying comments.

For SRL, courses can include goal-setting templates, calendar prompts, progress dashboards, brief weekly self-monitoring checks, and post-task reflection questions. These supports should gradually fade so that students assume greater control.

For social presence, interaction should be purpose-driven rather than simply increased. Small groups can receive rotating roles, evidence-based discussion prompts, deadlines, and explicit products. This design may reduce superficial interaction and connect social participation with regulation and inquiry.

For KSI, instructors can normalize question asking, provide optional resource pathways, ask students to generate inquiry questions, create low-stakes research extensions, and offer structured channels for peer and instructor help seeking. Students with lower KSI may need explicit prompts and accountability, whereas students with higher KSI may benefit from autonomy and choice.

### Limitations and future research

First, the cross-sectional design cannot establish temporal sequence or causality. Longitudinal and experimental designs are needed to test whether changes in CoI presences precede changes in SRL and outcomes.

Second, all constructs were measured with one self-report questionnaire. The high full-collinearity VIFs indicate that common method variance and multicollinearity cannot be ruled out.

Third, PTP is perceived rather than objective task performance. Future research should validate it against grades, task quality, completion records, or instructor ratings.

Fourth, the negative SP coefficient may reflect suppression, overlap, or model misspecification. Future research should compare alternative higher-order and sequential CoI models.

Finally, the significant moderated-mediation indices were probed with conditional point estimates at low, mean, and high KSI. Future studies should also examine other theoretically relevant moderators, including prior knowledge, self-efficacy, digital literacy, and epistemic beliefs.

## Conclusion

Overall, the findings largely support previous CoI and online-SRL research: SRL was positively associated with perceived task performance and online learning satisfaction, and TP and CP were positively related to SRL (Broadbent and Poon, 2015; Caskurlu et al., 2020; Shea and Bidjerano, 2010). The negative partial SP coefficient, however, contradicts the generally positive social-presence literature (Richardson et al., 2017; Hu et al., 2023) and is best treated as a model-specific suppression or collinearity result rather than a substantive harmful effect. KSI appears to condition the TP and SP pathways but not the CP pathway, extending CoI research by specifying a motivational boundary condition. At the same time, the marginal discriminant-validity violation, elevated collinearity diagnostics, same-source measurement, and cross-sectional design substantially limit confidence in the unique coefficients. The study’s most defensible contribution is therefore qualified: CoI resources, self-regulatory processes, and knowledge-seeking motivation should be examined together, but stronger measurement, verified conditional effects, longitudinal designs, and multi-source outcomes are required before causal or policy conclusions are drawn.

## Data Availability

The raw data supporting the conclusions of this article will be made available by the authors, without undue reservation.
