# Supplementary material for: Community of inquiry presences and undergraduates’ online learning outcomes: self-regulated learning as a pathway and knowledge-seeking intention as a boundary condition
Source: Front Psychol. 2026 Aug 19;17:1895876. doi: 10.3389/fpsyg.2026.1895876 (PMC13533949; doi:10.3389/fpsyg.2026.1895876)
Supplement: Supplementary file 1 [file Supplementary_file_1.docx]

**Appendix A**

# The administered instrument and analytic coding

The questionnaire contained 47 substantive items. Items 1-31 and 37-47 formed the 42-item analytic instrument. Items 32-36 were administered as a supplementary positive-affect block but were excluded from all measurement, structural, mediation, and moderation analyses reported in this article. All items used a five-point agreement scale from 1 (strongly disagree) to 5 (strongly agree).

Table A1

*Item-to-construct coding and analysis status*

| Original item numbers | Item block | Analytic assignment | Status in reported analyses |
| --- | --- | --- | --- |
| 1-7 | Teaching-presence items | TP | Included |
| 8-13 | Social-presence items | SP | Included |
| 14-19 | Cognitive-presence items | CP | Included |
| 20-26 | Self-regulated-learning items | SRL | Included |
| 27-31 | Knowledge-seeking-intention items | KSI | Included |
| 32-36 | Positive-affect items | None | Excluded from all reported analyses |
| 37-41 | Planning/task-performance items | PTP | Included |
| 42-47 | Online-learning-satisfaction items | OLS | Included |

*Note: The original item numbers correspond to the administered questionnaire. The 42 included items were used consistently in SPSS, SmartPLS, and PROCESS analyses.*

## Teaching presence (TP)

1. The teachers explained the online course objectives to the class.

2. The teachers informed the class about the deadlines and schedules for course learning activities.

3. The teachers helped the class understand the online course content.

4. The teachers encouraged the class to explore new concepts in the courses.

5. The teachers guided the class in participating in course learning activities.

6. The teachers provided timely feedback to the class.

7. The teachers assisted the class in completing online course learning tasks.

## Social presence (SP)

8. I felt as though the teachers were by my side.

9. Getting to know others gave me a sense of belonging in the class.

10. I found online communication to be an excellent medium for social interaction.

11. I felt that the class was a group that could communicate freely.

12. I felt comfortable using the online medium for conversation.

13. I was able to form distinct impressions of the online course participants.

## Cognitive presence (CP)

14. Combining new information helped me answer questions raised in course activities.

15. Reflecting on course content and discussions helped me grasp fundamental concepts.

16. I could apply the knowledge gained in online courses to my work or other activities outside class.

17. I felt motivated to explore content-related questions in online classes.

18. I utilized various information sources to explore problems posed in online courses.

19. Online discussions helped me appreciate different perspectives.

## Self-regulated learning (SRL)

20. I set standards for completing online learning tasks.

21. I set goals for my online learning tasks.

22. I had a daily schedule for my online learning activities.

23. I completed my online learning tasks according to my schedule.

24. I sought help from my peers when necessary.

25. I sought help from my instructors when necessary.

26. I regularly self-evaluated my progress in online learning.

## Knowledge-seeking intention (KSI)

27. I aimed to acquire knowledge through my current degree program.

28. I aimed to acquire knowledge through the offered courses.

29. I aimed to acquire knowledge through academic reading.

30. I aimed to acquire knowledge through academic writing.

31. I aimed to acquire knowledge through academic discussions and interactions.

**Supplementary positive-affect items (excluded from the reported analyses)**

32. I feel happy about the things I have to deal with.

33. I feel content with the tasks I have to do.

34. I am happy overall.

35. I laugh and feel relaxed often.

## 36. I feel like something good is going to happen.

## Perceived task performance (PTP)

37. I planned my online learning tasks to ensure they were completed on time.

38. I set priorities to complete my online learning tasks efficiently.

39. I focused on the outcomes I needed to achieve in my online learning.

40. I was able to distinguish between main issues and side issues in my online learning.

41. I could complete my online learning tasks efficiently with minimal time and effort.

## Online learning satisfaction (OLS)

42. I felt satisfied with my interaction with peers in online courses.

43. I was satisfied with the learning environment of online courses.

44. I was satisfied with the discussions with my peers in online courses.

45. I was satisfied with my relationships with my peers in online courses.

46. I was satisfied with the collaboration with my peers in online courses.

47. I was satisfied with the overall delivery of online courses.
